# A future classroom lab with active and gamified STEAM proposal for mathematics and science disciplines: Analyzing the effects on pre-service teacher’s affective domain

**DOI:** 10.1016/j.heliyon.2024.e35911

**Published:** 2024-08-20

**Authors:** Ana Isabel Montero-Izquierdo, Jin Su Jeong, David González-Gómez

**Affiliations:** Department of Experimental Science and Mathematics Education, Teacher Training College, University of Extremadura, Avd. de la Universidad s/n, Cáceres, 10004, Spain

**Keywords:** Future classroom Lab, STEAM, Mathematics and science didactics, Gamification, Affective domain

## Abstract

The recent emergence of innovative learning spaces, Future Classroom Lab (FCL), provides educators the use of physical learning spaces (to research, interact, exchange, develop, create, and present) and diverse technological tools to work according to active methodologies. Learners become more active in the learning process with the introduction of innovative learning environments that enable the application of interdisciplinary STEAM methodology and foster the development of 21st century competences. This study aims to uncover the probable link between application active and gamified STEAM educational interventions in the FCL and Pre-Service Teachers’ (PSTs) affective domain. The findings obtained showed statistically significant variations and, therefore, positive effects on the PSTs’ affective domain (self-efficacy, attitude, and emotion) after performing the intervention. The sample consisted of a total of 54 PSTs enrolled in the second year of Primary Education. Limited studies regarding the affective domain in the FCL were found, which restricted the comparison with prior research. This study has several implications, such as the introduction of innovative educational proposals to PSTs at the university level and, consequently, the implementation of similar interventions in elementary schools. This research intended to reveal how the different variables work as a support system for students’ learning process in mathematics and science disciplines.

## 1 Introduction

In recent years, there has been a noticeable increase in the use of innovative learning environments, active methodologies, and interdisciplinary education to enhance students’ learning processes. The dynamic interplay between pedagogy and spatial design facilitates students’ acquisition of 21st century skills and successful learning [1]. The Future Classroom Lab (FCL) is a space that favors competency-based learning and provides diverse learning areas for students to investigate, interact, exchange, develop, create, and present [[2], [3], [4]]. The FCL invites educators to experiment and rethink the different teaching-learning methodologies, as well as to promote the application of active and gamified approaches [[5], [6], [7]]. With the introduction of new learning environments, where each place resembles the center of the classroom, students become more engaged, motivated, and involved in their learning process [8]. Furthermore, the FCL aims to provide educators with a learning space where they can acquire the necessary skills to teach in this type of classroom [4,9]. One indicator of a favorable learning environment is well-being, a particular notion that integrates emotional and cognitive elements [10]. When good feelings and thoughts about school, teachers, classmates, and the entire educational environment predominate over negative ones, students can progress toward their academic and social goals and enjoy a positive school experience [11]. Several studies also revealed that learner-centered methodologies generate more positive emotions than traditional approaches [12,13].

Over the last decades, many authors have questioned the effectiveness of traditional methodologies and defended the need for students’ self-construction of knowledge [14,15]. The use of active learning methodologies engages and motivates students in their learning process in contrast to traditional teaching methods [[16], [17], [18]]. Gamification is a type of active methodology and is considered a powerful tool for teachers at all levels of the educational system [19,20]. Many authors have identified that students’ motivation can increase when gamification is implemented correctly, which contributes to their engagement in learning activities [[21], [22], [23]]. According to Asunción [24], active methodologies promote the development of students’ skills, such as autonomy, participatory attitude, communication, cooperation, problem solving, creativity, and others for the construction of knowledge. The European Schoolnet considers Science, Technology, Engineering, and Mathematics (STEM) education as one of the main areas of research within the FCL project and develops a wide range of actions aimed at promoting this relatively new approach within education [5]. This is consistent with the fact that many jobs requiring STEM skills are being created [7].

Science, Technology, Engineering, Arts, and Mathematics (STEAM) education is also one of the most impressive educational movements in recent years [[25], [26], [27]]. Some authors agree that adding arts means incorporating critical thinking, problem-solving tasks, and the ability to apply arts into real-life situations, where students can use their analytical and creative minds to solve complex problems [28]. STEAM was proposed as a response to students’ need to succeed in understanding the connections between disciplines [29,30]. Interdisciplinary STEAM education is aligned with 21st century pedagogy [31,32] and aims to improve human competencies in the 21st century [[33], [34], [35]]. According to literature, the implementation of STEAM proposals had demonstrated to have a positive impact on students’ cognitive and emotional learning [36,37]. In addition, STEAM experiences positively affected the emotive domain [36,38,39] and enhance students’ learning motivation, self-efficacy [[40], [41], [42]] and academic performance [40]. Perceived self-efficacy is an essential component of memory functioning, impacting cognitive, emotional, and motivational processes [43]. In addition, STEAM-based activities greatly increase students’ achievement and attitude [44].

The feeling or emotional experience is called affect [45]. Emotions are crucial for learning and success because of their intimate links to behavioral, cognitive, motivational, and physiological processes [46]. The main basic components of the affective domain are self-efficacy, attitude, and emotion, and they are of great importance in teaching-learning processes [47]. Traditionally, mathematics and science education has focused more on cognition than on the affective domain [48]. However, affective domain and the teaching-learning process are inextricably linked [47,49]. Moreover, enhancing affective domain has a positive impact on students’ academic achievements [11,50]. Boredom, indifference, and difficulty cause students to abandon their mathematical and scientific vocations when choosing courses or jobs [[51], [52], [53]]. The application of innovative learning settings, along with the effective use of technology and active methodologies, has led to an improvement in students’ attitudes toward learning experiences [54]. In addition, integrated course design boosted students’ learning motivation, self-efficacy, and interdisciplinary knowledge acquisition [41]. A significant number of students attribute this change in emotional predisposition to increased self-efficacy [55,56]. Also, Elliott [57] observed that integrated methodologies increased students’ interest in STEM subjects and that there was a favorable association between students’ attitudes and performance. In general, a favorable emotional environment improves the quality of the learning process since emotion is linked to the capacity for attention and information storage [49].

Therefore, having positive affective domain is crucial for the learning processes. However, few research studies have been found related to active and gamified STEAM proposals in the FCL that analyze PSTs’ affective domain. For this reason, this study aims to evaluate the influence of the intervention on PSTs in terms of affective domain: (1) self-efficacy toward mathematics, science and the FCL (2) attitude toward the FCL, and (3) emotion toward the intervention.

## 2 Materials and methods

In this research, an active and gamified STEAM proposal has been performed within the FCL. It consisted of an interdisciplinary intervention within the subjects ‘Mathematics and its Didactics’ and ‘Matter and Energy’ in the FCL. The instruments used for data collection were pre- and post-questionnaires with a 5-point Likert-type scale. The results obtained were analyzed through various statistical analyses, including descriptive statistics, non-parametric and reliability analysis.

### 2.1 Sample and course context

The study was conducted with a total of 54 PSTs, enrolled in the second year of Primary Education degree, during the second semester of the 2021/2022 academic year. The sampling method used for this research is convenience sampling which is a non-probability sampling method. The demographic information of the PSTs is shown in Table 1. The participants in this study had a mean age of 20.2 years old, of which 60 % were women and 40 % were men. The PSTs’ educational background were Social Sciences or Humanities (52 %), Science (37 %), Technology (7 %) and others (4 %).

**Table 1:** Demographic information about age, gender, and pre-university educational background of the PSTs.

N	Age	Gender (%)		Pre-university educational background (%)			
54	20.2	Male	Female	Social Science or Humanities	Science	Technology	Others
		40	60	52	37	7	4

In relation to the subject of ‘Mathematics and its Didactics’, the topics involved in this intervention were numeration systems and arithmetic operations. Regarding the subject of ‘Matter and Energy’, the topic of the universe was chosen for this educational proposal. Table 2, Table 3 provide specific information on the teaching plan of the subjects involved in the intervention are provided.

**Table 2:** Course Description about the Teaching Plan of ‘Mathematics and its Didactics’.

Subject: Mathematics and its Didactics		
Topic	Course context Description	Hours
Natural numbers and numeration systems	Mathematical science	25/150
	Coordinate sets	
	Concept of number	
	Positional number system	
	Decimal numeration	
	Teaching strategies and resources	
Arithmetic operations	Meaning and mechanism of operations	25/150
	Additions and subtractions without and with compensation	
	Multiplications without and with regrouping	
	Divisions with one and several figures	
	Potentiation and nth root	
	Problem solving	
	Teaching strategies and resources

**Table 3:** Course Description about the teaching plan of ‘matter and energy’.

Subject: Matter and Energy		
Topic	Course context Description	Hours
The Universe	The size of the Universe	30/150
	Origins and evolution of the Universe	
	The Universe's fundamental structures: galaxies	
	The stars and planetary systems	
	The Solar System	
	Didactic sky models for Primary School Education	

### 2.2 Data statistical analysis

The influence on affective domain (self-efficacy, attitude, and emotion) of the PSTs was measured through pre- and post-questionnaires. The reliability and the internal consistency of the instrument was assessed by means of Cronbach’s alpha. The results were obtained for each scale (self-efficacy items alpha = 0.90, attitude items alpha = 0.88, and emotion items alpha = 0.92). In addition, the scale reliability of the whole instrument was also assessed (alpha = 0.93), showing a good internal and reliable consistency. On the other hand, a Kolmogorov-Smirnov test was used to test the normality of the dependent variables self-efficacy (W = 0.879; p < 0.001), attitude (W = 0.814; p < 0.001), and emotion (W = 0.927; p < 0.001). Based on these results, a non-normal distribution was assumed and, therefore, nonparametric tests were used to assess the effects of the intervention in students’ self-efficacy, attitude, and emotion [58]. Hence, the Mann-Whitney was applied to compare the mean effects, and in those cases where significant differences were detected, the effect sizes were calculated using the rank-biserial correlation (r_rb_) for non-parametric data [59,60]. The statistical analysis was conducted by using Jamovi, an open-source software built on the R statistical language [61]. In addition, Microsoft Excel was employed for data visualization and plotting of data analyzed.

### 2.3 An active and gamified STEAM activity within the FCL

**Fig. 1:**
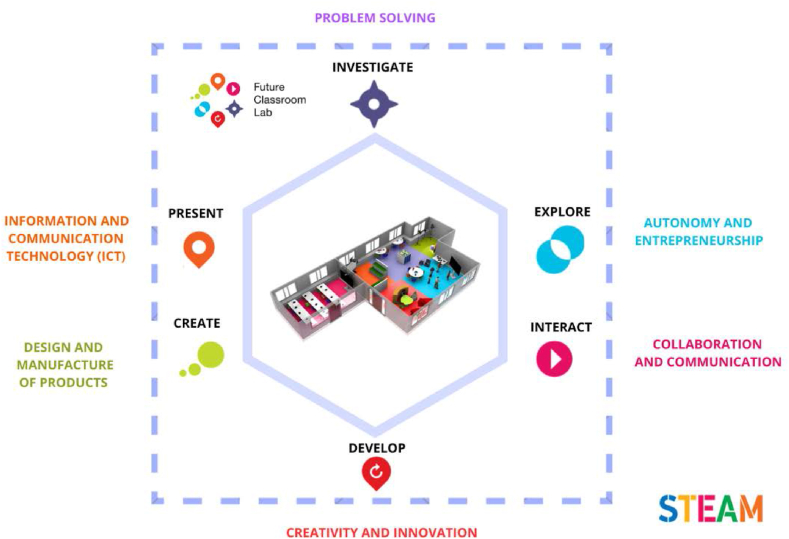
Different learning zones of the FCL adapted from European Schoolnet [5] and STEAM competences adapted from Ludeña [62].

The intervention consisted of an active and gamified STEAM learning experience within the FCL. For the design of this educational proposal, mathematics and science subjects were considered and the specific topics involved were the numeration system and the universe. Several activities were proposed in the different learning areas of the FCL. This innovative classroom offers diverse working zones, such as investigate, create, develop, interact, present, and interchange. These learning areas are designed to be used according to students’ needs and permits the teacher to act as a facilitator, providing feedback to the learners. Also, classroom setting promotes collaborative work and the development of competences (see Fig. 1), such as problem-solving, autonomy, creativity, innovation, digital competence, and collaboration [62].

**Fig. 2:**
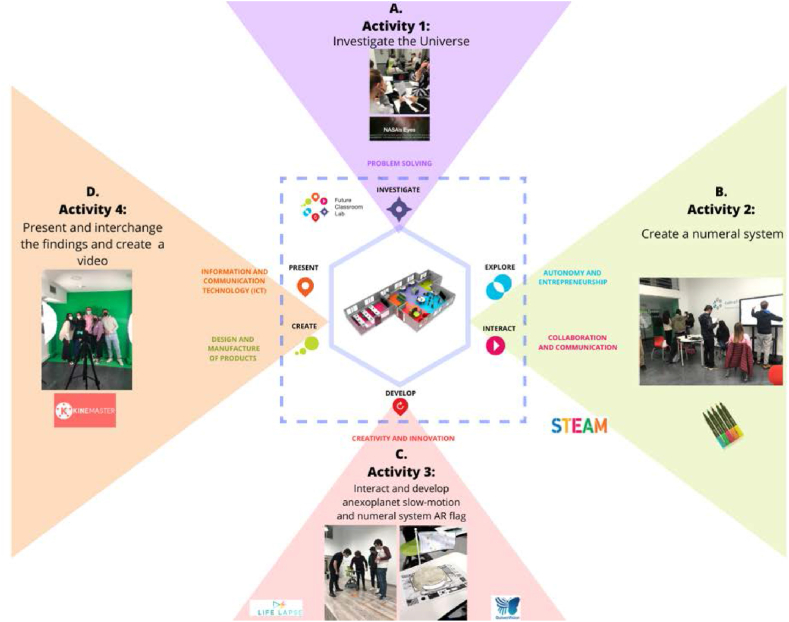
Activities conducted in the intervention according to the different spaces and competences developed.

The intervention includes four main activities: (1) Investigate the universe, (2) Create a numeration system, (3) Interact and develop an exoplanet slow-motion and an augmented reality flag with concerning the numeration system designed, and (4) Interchange and present the findings (see Fig. 2).

**Fig. 3:**
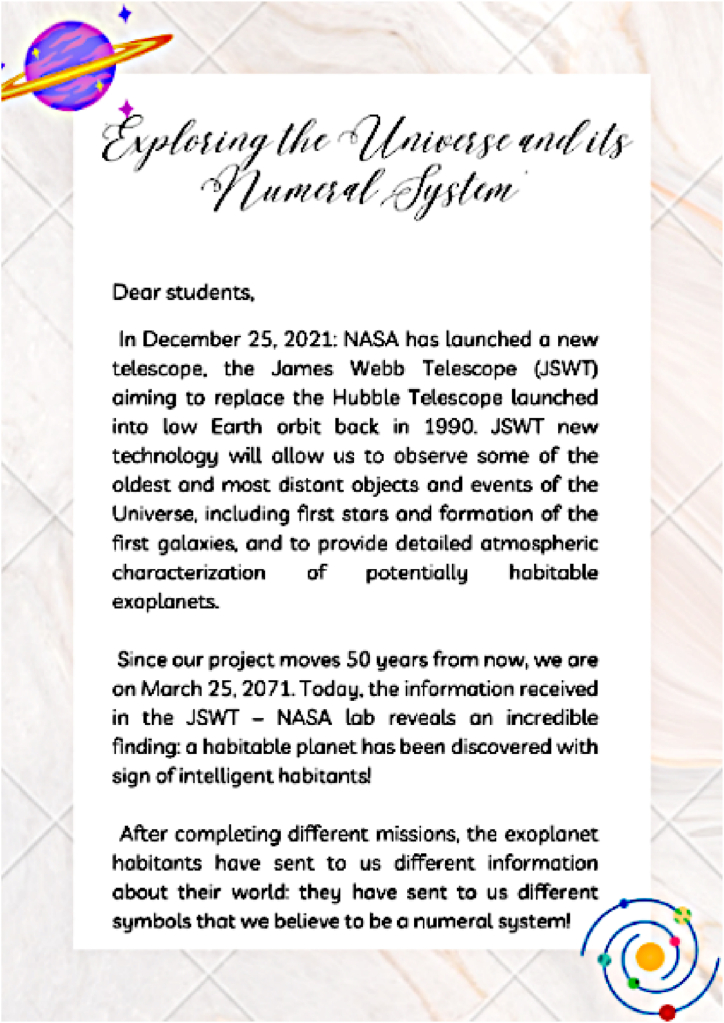
Engaging letter of the didactic intervention in the FCL.

First, to introduce the gamified didactic proposal, a captivating letter was delivered to the participants with the aim of providing a clear understanding of the intervention goals but in a gamified way by connecting the diverse activities around the narrative (see Fig. 3).

The participants were divided into small groups of five students and the activities were then conducted in various learning zones of the FCL. In terms of intervention, four main activities were created in each area (see Fig. 2). The duration of the entire intervention was 2 h in total and 30 min for each of the four activities. The initial activity was carried out in the ‘investigate’ learning area (see Fig. 2a). PSTs used the laptops and mobile phones to access NASA's official website to learn about the exoplanets. PSTs filled out a questionnaire ‘Eyes on Exoplanets’ and the ‘Planet Card’ (see Table 4). The second activity was held in the ‘create’ area. The aim was to construct a novel numeration system that could express any number consistently by creating new symbols (see Fig. 2b), and any numerical base could be chosen to create the design. In the end, PSTs had to perform different arithmetic operations (addition, subtraction, multiplication, and division) and establish a relationship between the numeration system created and the decimal number system. In summary, this activity required students to sketch the numeration system on a digital board and/or on a glass wall. Then, in the ‘develop’ and ‘interact’ areas, the PSTs had to make a slow-motion video of an exoplanet (see Fig. 2C). The aim of this activity was to illustrate how an exoplanet orbits its moon within the solar system. The next activity consisted of the creation of a symbolic augmented reality flag in relation to the numeration system created. The augmented reality application Quiver was used to obtain an augmented reality image and video (see Fig. 2c). Finally, in the ‘present’ and ‘exchange’ areas, the aim was to create a video using the chroma screen provided by the FCL. The PSTs were required to present all the discoveries made during the intervention and describe the results of each project activity (see Fig. 2d).

**Table 4:** ‘Eyes on exoplanets’ and ‘Planet Card’ questionnaires.

‘Eyes on Exoplanets’
• How many confirmed planets are in the Milky Way? •What is an exoplanet? •The habitable zone is the area around a star where it is not too hot and not too cold for liquid water to exist on the surface of surrounding planets •Do exoplanets have stars like our Sun? •When was Kepler-452 b discovered? •Scientists believe that Kepler-452 b can support life •How many days has Kepler 452byear?
‘Planet Card’
•Planet name •Distance from Earth (Light-years) •Date of Discovery •Type of Star Planet mass (compering to Earth) •Orbital Period •Orbital Radius •Is the planet inside the habitable zone? •Picture of the Planet •Picture of the Planet and Earth •Picture of the Planet solar system •Brief description of the planet

### 2.4 Instruments

Regarding with the instruments used in the study, pre- and post-online questionnaires were applied for data collection. The aim was to measure the self-efficacy, attitude, and emotion items of the PSTs before and after the intervention. Participation was voluntary and anonymous. Participants’ confidentiality was guaranteed, since the PSTs were informed through the questionnaire and accepted the bioethical consent. The Bioethics and Biosafety Committee of the University of Extremadura approved the bioethical consent, with registration numbers 94/2018 and 77/2023. The survey contained a total of 29 items of which 10 items referred to self-efficacy, 5 items to attitude, and 14 to emotion (see Table 5). A 5-point Likert-type scale was used in the questionnaire, ‘strongly disagreed’ (SD), ‘disagreed’ (D), ‘neutral’ (N), ‘agreed’ (A), and ‘strongly agreed’ (SA). The various items used to measure self-efficacy in this intervention were personalized from the Science Teaching Efficacy Belief Instrument (STEBI-B). The questionnaire used in relation to attitudes was modified with respect to those previously used in other studies [[63], [64], [65]]. Both questionnaires have been adapted from previous published and validated research [66] and the emotion questionnaire was adapted from Dunbar et al. [67].

**Table 5:** Questionnaire Description about Self-efficacy(SE), Attitude(AT), and emotion(EM) items.

Affective domain	Items
Self-efficacy toward mathematics, science and FCL	SE1. I understand math/science concepts well enough to teach math/science at the lower educational levels.
	SE2. I will usually be able to answer students' math/science questions.
	SE3. When I put all my efforts, I will succeed in teaching math/science as well as I would in other subjects.
	SE4. I believe I have the necessary skills to teach math/science.
	SE5. Math/science is useful for solving everyday problems.
	SE6. It is important to know math/science to get a good job.
	SE7. I know the steps necessary to teach math/science effectively.
	SE8. I encounter difficulties when trying to explain a mathematical/scientific concept.
	SE9. The use of motivating teaching spaces is essential to achieve good learning results.
	SE10. I know how to work in the FCL.
Attitude toward FCL	AT1. I prefer a FCL to a traditional theory class to teach math and science content.
	AT2. I prefer a FCL to a traditional lab session to teach math and science content.
	AT3. Working on the contents of several subjects simultaneously favors learning.
	AT4. Working in a FCL type environment enhances creativity in students.
	AT5. Working in a FCL type environment improves student collaboration.
Emotion toward the intervention	EM1. Joy
	EM2. Satisfaction
	EM3. Enthusiasm
	EM4. Fun
	EM5. Trust
	EM6. Hope
	EM7. Pride
	EM8. Uncertainty
	EM9. Nervousness
	EM10. Concern
	EM11. Frustration
	EM12. Boredom
	EM13. Fear
	EM14. Anxiety

## 3 Results and discussion

To measure the impact of the intervention on PSTs’ affective domain, participants were requested to complete online questionnaires before and after the intervention on the three dimensions: self-efficacy, attitude, and emotion. The responses given by the participants in the 29-items questionnaire are shown in Fig. 5, Fig. 6. The Affective Domain Score (ADS) was calculated considering the sum of all the variables studied: Global Self-Efficacy Score (GSES), Global Attitude Score (GAS), and Global Emotion Score (GES).

### 3.1 Self-efficacy results

**Fig. 4:**
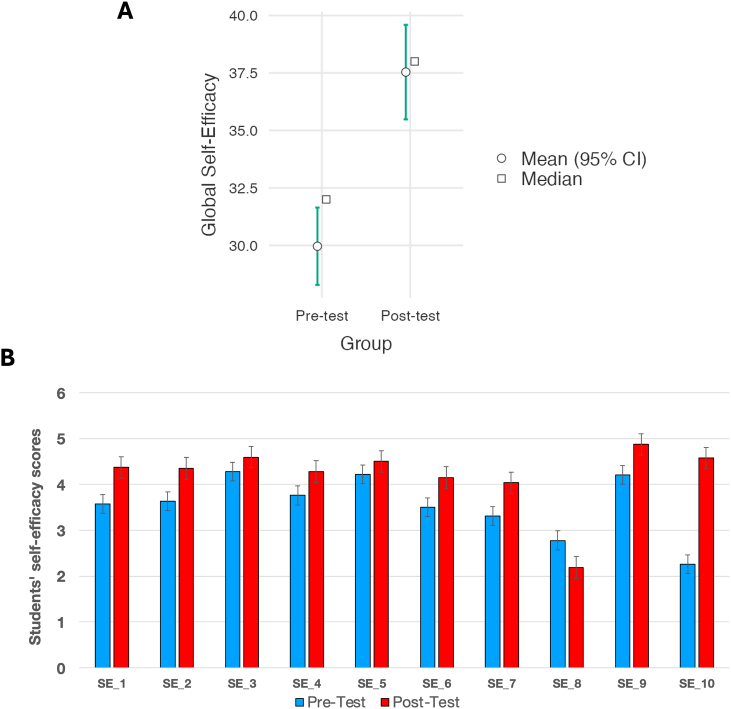
Comparison results of the self-efficacy globally calculated (A) and for each individual self-efficacy item (B).

Regarding the participants’ self-efficacy beliefs toward mathematics, science, and FCL, a GSES was calculated as the sum of each of the individual self-efficacy scores reported by the students $\left(GSES=\sum {SE}_{i}\right)$. The comparison of the results indicated that significant differences were observed after completion of the intervention (U = 559; p < 0.001; *r*_*rb*_ = 0.62; *CI*_95_[6.0,11.0]) and according to these results, the effect obtained was very high. Fig. 4a summarizes the comparison of the GSES before and after the intervention, and this figure also includes the comparison for each item individually (see Fig. 4b). The items from SE_1 to SE_8 refer to self-efficacy toward mathematics and science and SE_9 and SE_10 toward the FCL.

**Fig. 5:**
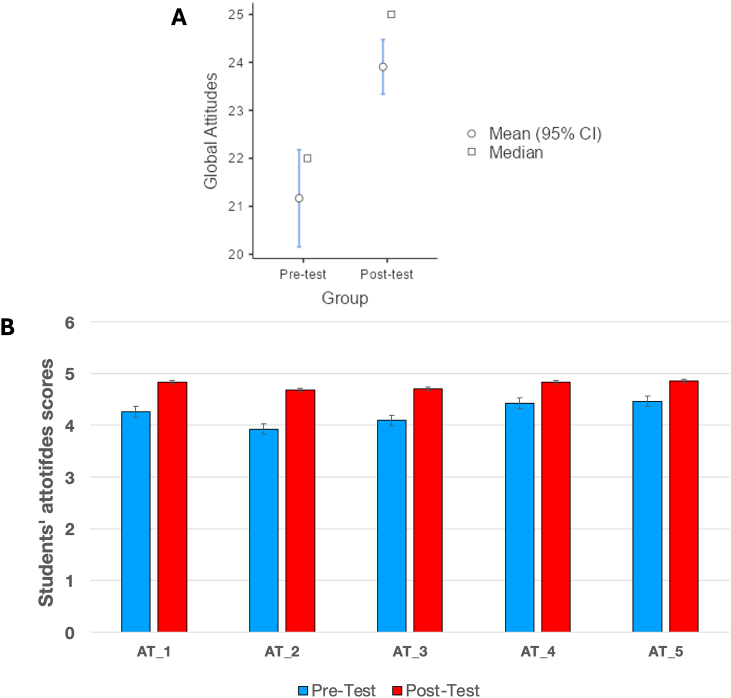
Comparison results of the attitude globally calculated (A) and for each individual atitude item (B).

**Fig. 6:**
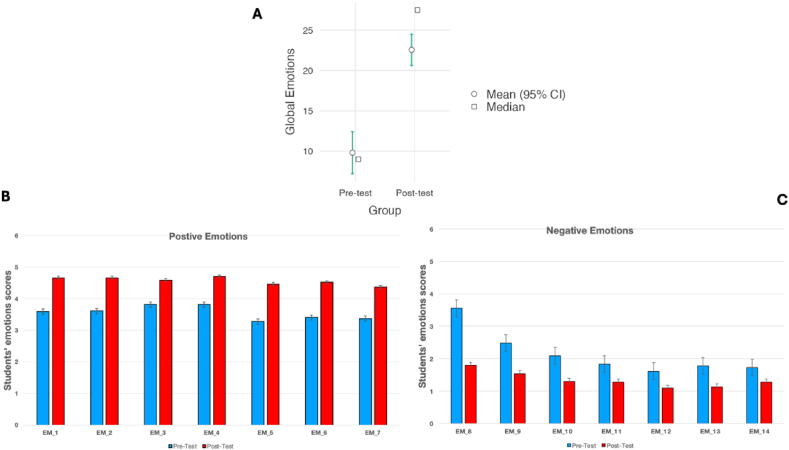
Comparison results of the emotions globally calculated (A) and for each individual positive (B) and negative (C) emotion item.

In addition, the influence of students’ educational background was assessed, resulting in a significant increase in all cases, with the exception of the Technology background (U = 161; p < 0.001; *r*_*rb*_ = 0.61; *CI*_95_[5.0,12.0] for Social Sciences background; U = 65.0; p < 0.001; r_rb_ = 0.65; *CI*_95_[4.0,11.0] for Science background and U = 3.5; p = 0.133; *CI*_95_[4.0,12.0] for Technology background).

### 3.2 Attitude results

Regarding the results of attitudes toward the FCL, a GAS was calculated as a sum of each individual attitude item $\left(GAS=\sum A_{i}\right)$. According to the results, a significant increase in participants’ attitude was observed after completing the intervention (U = 690; p < 0.001; *r*_*rb*_ = 0.57; *CI*_95_[1.0,3.0]). In this case, the results on effect size revealed a genuine medium effect (see Fig. 5a). Regarding each individual attitude item, a significant increase was observed in all of them after the intervention (see Fig. 5b).

Finally, the data obtained were also analyzed considering the students’ educational background. In all cases, scores increased significantly, except in the case of students with a Technology background, where the increase was not significant (U = 208; p < 0.001; *r*_*rb*_ = 0.49; *CI*_95_[0.0,3.0] for Social Sciences background; U = 94.0; p = 0.006; *r*_*rb*_ = 0.51; *CI*_95_[1.0,4.0] for Science background and U = 3.5; p = 0.106; *CI*_95_[0.0,6.0] for Technology background).

### 3.3 Emotions results

The students’ emotions toward the educational intervention were assessed. Since the scale used in the instrument showed high internal consistency, a GES was calculated as $GES=\sum \left({Positive}_{emotions}-{Negative}_{emotions}\right)$. The mean values of the emotions scores before the intervention was 9.81 (std dev = 9.74) and after the intervention 22.6 (std dev = 7.28). Comparison of the GES before and after the intervention revealed a significant increased (U = 385; p < 0.001; *r*_*rb*_ = 0.74; *CI*_95_[9.0,16.0]). In this case, the effect size suggested that there was a genuine difference (very high effect) in students’ emotions after the teaching intervention (see Fig. 6a). Additionally, all the emotions (see Fig. 6b and c), showed significant differences (p < 0.001).

The influence of students’ educational background on emotion was also assessed, and it was observed there was significant difference in GES for all educational background according to the effect size values, but not for those with a Technological background (U = 91.5; p < 0.001; *r*_*rb*_ = 0.78; *CI*_95_[8.0,20.0] for Social Sciences background; U = 71.0; p < 0.001; r_rb_ = 0.63; *CI*_95_[5.0,15.0] for Science background and U = 2.0; p = 0.055; *r*_*rb*_ = 0.80; *CI*_95_[4.0,27.0] for Technology background).

### 3.4 Affective domain results

Finally, in order to assess how the active and gamified STEAM intervention in the FCL influenced the participants’ affective domain as a whole, ADS was calculated considering all the variables studied ($ADS=\sum {SE}_{i}+\sum {AT}_{i}+\sum \left({Positive}_{emotions}-{Negative}_{emotions}\right)$. When comparing ADS before and after the intervention, statistically significant differences were observed (U = 379; p < 0.001; *r*_*rb*_ = 0.75; *CI*_95_[18.0,28.0]). The mean score values of ADS scores before the intervention were 60.9 (std dev = 16.1) and after the intervention 84.0 (std dev = 15.1). In addition, the effect size values showed that this difference was genuine (very high effect). Considering participants’ educational background, it was found that for all educational backgrounds the effect size values were significant in relation to ADS, except for the Technological background (U = 112; p < 0.001; *r*_*rb*_ = 0.73; *CI*_95_[16.0,34.0] for Social Sciences background; U = 55.0; p = 0.006; *r*_*rb*_ = 0.71; *CI*_95_[12.0,27.0] for Science background and U = 3.0; p = 0.105; *CI*_95_[4.0,14.0] for Technology background).

## 4 Discussion

Upon the completion of the active, gamified and STEAM innovative proposal in the FCL and according to the data analysis, significant differences were obtained in terms of the PSTs’ affective domain (self-efficacy, attitude, and emotion).

First, the self-efficacy of PSTs toward mathematics and science was explored. The results are consistent with the findings of Jia et al. [41] and Kong & Huo [42], who mentioned that STEAM experiences positively influenced the improvement of students’ learning motivation and self-efficacy. Given the significant influence that self-efficacy has on academic achievement and students’ achievement in STEM fields [68,69], it is essential to offer experiences that strengthen students’ sense of self-efficacy. In addition, providing students with the opportunity for hands-on research experiences can have a significant impact on fostering an environment in which students can build a foundation of scientific understanding and skill set, which can help students enhance their self-perception as scientists [70]. Understanding and correctly applying mathematics and science is crucial for students to succeed academically in STEM fields. Practical approaches, such as ‘Using and Doing’ science, involve the application of scientific knowledge and skills to address problems in practical contexts and have a positive impact on self-efficacy and self-confidence about mathematical and scientific knowledge and skills [71]. According to Günes [72], PSTs’ perception of their own self-efficacy and mathematical competence is shaped by previous experiences. Therefore, high levels of perceived mathematical self-efficacy can enhance PSTs’ learning effectiveness.In terms of self-efficacy towards FCL, the results showed significant differences. These findings are consistent with those of other authors who mentioned that the implementation of educational interventions in the FCL enhanced educators’ self-efficacy, as it provides a testing ground to develop the skills needed to teach in this type of classroom [4,9]. Additionally, when each learning zone resembles the center of the classroom, learners become more interested, motivated, and involved in the learning process [8]. Furthermore, increasing self-efficacy is important, since teachers’ self-efficacy, attitude, and students’ educational background have a considerable impact on their mathematical learning [73].

Regarding attitude, when compared to other academic disciplines, students show more adverse attitudes toward mathematics and science [74]. According to the results, students’ attitudes toward the FCL learning environment improved significantly after the intervention. Elliott [57] discovered that integrated approaches boosted students’ interest in STEM courses and that there was a positive relationship between students’ attitudes toward mathematics and their performance. Moreover, the introduction of curriculum integration has been shown to enhance PSTs’ effectiveness and confidence [[75], [76], [77]]. Likewise, the use of innovative learning environments and active methodologies has been shown to improve students’ attitudes toward educational experiences [54]. According to Park & Choi [8], active learning classrooms promote more inspiring educational practices, encourage active participation, and reduce the gap in attitudes toward learning between high- and low-achieving students. In addition, some authors found positive associations between the learning environment, motivation, and students’ attitudes toward mathematics [[78], [79], [80], [81]]. These findings are relevant as students perform better when they are motivated and have a pleasant learning environment [80].

Regarding the emotion and affective domain toward the intervention, significant results were obtained. Learning environments have been shown to foster favorable emotional experiences that lead to a variety of emotional, cognitive, and behavioral changes and developments, as well as increased personal engagement in the learning process [82]. Active learning environments have been shown to have a positive impact on students, greatly improving students’ emotions, self-confidence, and beliefs about mathematics [80]. Also, emotions play an important role in driving learning in technology-based learning environments, and the characteristics of these environments can influence students’ affective experiences [83,84]. The affective domain is strongly tied to the teaching-learning process and is considered a key piece to provide a successful learning process [47,49]. In addition, STEAM experiences were found to positively influence the affective domain [36,38,39]. It has also been uncovered that the application of learner-centered approaches generates more pleasant emotions than traditional methods [12,13].

The application of the active, gamified and STEAM innovative proposal in the FCL as a learning environment has positively impacted on PSTs’ affective domain, which revealed how the different variables can act as a support system for students’ learning in mathematics and science disciplines. Likewise, regarding the relationship between the PSTs’ educational background and each affective domain component (self-efficacy, attitude, and emotion), significant values of effect sizes were observed concerning Social Sciences or Humanities and Sciences background.

## 5 Conclusions

According to the overall results, the application of the active and gamified STEAM proposal in the FCL had a positive influence on PSTs’ affective domain (self-efficacy, attitude, and emotion). This research established a theoretical value as it provided an appreciable synthesis of theoretical aspects of previous studies and new explanatory variables that can enhance teaching-learning processes in mathematics and science courses. Moreover, the importance of this research has been proved to have great social and practical value, as it has the potential to serve as a model for non-experienced PSTs, improving their professional practice. Therefore, this research has various implications for educational innovation, including the introduction to PSTs of novel learning strategies through the educational proposal and, the execution of analogous interventions in elementary schools. Furthermore, this study can contribute to raising teachers' awareness of the importance of the affective domain in effective learning in mathematics and science. In relation to the reliability and validity of the instruments obtained, this research can provide replicability of the intervention for future research. Nevertheless, the limited number of studies on PSTs’ affective domain through STEAM methodology in the FCL, makes it difficult to compare with previous research. Finally, this research serves as a pioneering study to explore the possible relationship of PSTs’ affective domain regarding the application of an active and gamified STEAM intervention in the FCL, innovating in the field of mathematics and science didactics.

## Availability of data and materials

The datasets used and/or analyzed during the current study are available from the corresponding author on reasonable request.

## Funding

No funding was received for conducting this study.

## CRediT authorship contribution statement

**Ana Isabel Montero-Izquierdo:** Writing – review & editing, Writing – original draft, Visualization, Validation, Software, Methodology, Investigation, Formal analysis. **Jin Su Jeong:** Writing – review & editing, Writing – original draft, Visualization, Validation, Supervision, Software, Resources, Project administration, Methodology, Investigation, Funding acquisition, Formal analysis, Data curation, Conceptualization. **David González-Gómez:** Writing – review & editing, Writing – original draft, Visualization, Validation, Supervision, Software, Resources, Project administration, Methodology, Investigation, Funding acquisition, Formal analysis, Data curation, Conceptualization.

## Declaration of competing interest

The authors declare that they have no known competing financial interests or personal relationships that could have appeared to influence the work reported in this paper.
