# Supplementary material for: A future classroom lab with active and gamified STEAM proposal for mathematics and science disciplines: Analyzing the effects on pre-service teacher’s affective domain
Source: Heliyon. 2024 Aug 20;10(16):e35911. doi: 10.1016/j.heliyon.2024.e35911 (PMC11382054; doi:10.1016/j.heliyon.2024.e35911)
Supplement: Multimedia component 1 [file mmc1.pdf]

Questionnaires

Background information:

Gender:
Age:
Educational Background:
University Access:

Please select 1 to 5 (1: totally disagree to 5: totally agree)

Questions of Self-Efficacy Items:

SE_1: I understand math/science concepts well enough to teach math/science at the lower educational levels.
SE_2: I will usually be able to answer students' math/science questions.
SE_3: When I put my all into it, I will succeed in teaching math/science as well as I would in other subjects.
SE_4: I believe I have the necessary skills to teach math/science.
SE_5: Math/science is useful for solving everyday problems.
SE_6: It is important to know math/science to get a good job.
SE_7: I know the steps necessary to teach math/science effectively.
SE_8: I encounter difficulties when trying to explain a mathematical/scientific concept.
SE_9: The use of motiving teaching spaces is essential to achieve good learning results.
SE_10: I know how to work in a Classroom of the Future.

Questions of Attitude Items:

AT_1: I prefer a Classroom of the Future to a traditional theory class to teach math and science content.
AT_2: I prefer a Classroom of the Future to a traditional lab session to teach math and science content.

AT_3: Working on the contents of several subjects simultaneously favors learning.
AT_4: Working in a future classroom type environment enhances creativity in students.

Questions of Emotion Items:

EM_1: Joy
EM_2: Satisfaction
EM_3: Enthusiasm
EM_4: Fun
EM_5: Trust
EM_6: Hope
EM_7: Pride
EM_8: Uncertainty
EM_9: Nervousness
EM_10: Concern
EM_11: Frustration
EM_12: Boredom
EM_13: Fear
EM_14: Anxiety

Thanks for your participation.
